# Guides for users and prescribers of lithium

**DOI:** 10.1186/s40345-019-0166-8

**Published:** 2019-12-23

**Authors:** Janusz K. Rybakowski

**Affiliations:** Department of Adult Psychiatry, Poznan University of Medical Sciences, Szpitalna 27/33, 60-572 Poznan, Poland

In their comprehensive and updated guide of clinical use of lithium, Tondo et al. (2019), also made a historical account of persons who significantly contributed to these guidelines. In this context, it would be appropriate to mention the recently deceased great American supporter of lithium therapy, James Walter (Jeff) Jefferson (1937–2019).

In 1975, Jeff Jefferson, together with his colleagues from the Madison University in Madison, Wisconsin, established the Lithium Information Center, aiming to organize and disseminate all emerging publications connected with lithium in medicine. In this sense, it was complementary to the efforts of the great European *spiritus movens* of lithium, Mogens Schou (Grof and Müller-Oerlinghausen 2018), who in 1969, initiated the lithium bibliography (Schou 1969), succeeded by eight publications in this respect, for the next 12 years (Schou 1981). The Center moved in 1998 to the Madison Institute of Medicine, and in 1999 was included into the Bipolar Disorders Treatment Information Center. In the recent fundamental lithium book “Lithium in Neuropsychiatry” which appeared in 2006, it was reported that the Center had a library of over 40,400 references indexed by over 9800 distinct subject/keyword headings that could be computer searched by title, word, author, journal and year of publication. Up to this time, the information specialists from the center have responded to over 45,000 requests from a wide range of individuals and organizations (Jefferson et al. 2006). As to the guidelines of lithium therapy, it should also be noted that Jeff Jefferson was the principal author of the first American lithium handbook titled “Lithium Encyclopedia in Clinical Practice” which was published in 1984 (Jefferson et al. 1984), and the second edition of the encyclopedia appeared in 1987.

Jeff Jefferson was also greatly engaged in the important topics of lithium administration. In 1998, in the *British Medical Journal* (Jefferson 1998), he formulated a strong rebuttal against the articles of Joanna Moncrieff who expressed a skepticism for therapeutic and prophylactic efficacy of lithium (Moncrieff 1995) As the kidney issue of lithium therapy became of great importance in recent years, he published a clinician guide to monitoring kidney function in lithium-treated patients (Jefferson 2010). I remember him from numerous lithium conferences, as an extremely friendly person and sincerely committed to the promulgation of lithium therapy.

It can be considered that, given the importance of lithium for the treatment of bipolar, and—often forgotten—unipolar mood disorders (Abou-Saleh et al. 2017) and on the other side, underutilization of its use globally, an Information Center similar to what Jefferson built up, would be much needed in these days. The only scientific group in the world supporting lithium nowadays is the International Group for the Study of Lithium-Treated Patients (IGSLI) http://www.igsli.org. Most recently, the eminent specialist on bipolar disorder, Robert Post (2018) deplores that lithium is underutilized in the USA, even to a greater extent than in European countries. He points to the multiple assets of lithium beyond its antimanic and prophylactic effect, such as antidepressant action, suicide prevention, pro-cognitive and anti-dementia effects as well as diminishing the frequency of a few medical conditions. In line with this, the author of this letter in his recent paper focuses on challenging the negative perception of lithium and optimizing its long-term administration (Rybakowski 2018).

Coming back to the Tondo et al.’s paper it should not go unnoticed that in the first sentence of the paper, the dates of John Cade were put incorrectly, as this introducer of lithium therapy 70 years ago was born in 1912 and passed away in 1980.

## Data Availability

Not applicable
